# How Does the Antibiotic Amphotericin B Enter Membranes and What Does It Do There?

**DOI:** 10.1021/acs.jpclett.4c00496

**Published:** 2024-04-26

**Authors:** Sebastian Janik, Rafal Luchowski, Ewa Grela, Wojciech Grudzinski, Wieslaw I. Gruszecki

**Affiliations:** † Department of Biophysics, Institute of Physics, Maria Curie-Sklodowska University, 20-031 Lublin, Poland; ‡ Department of Biophysics, Medical University of Lublin, 20-059 Lublin, Poland; § Division of Biophysics, Institute of Experimental Physics, Faculty of Physics, University of Warsaw, 02-093 Warsaw, Poland

## Abstract

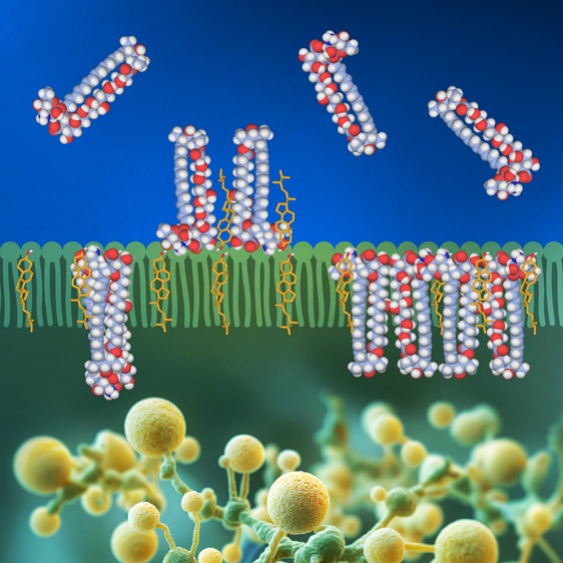

Amphotericin B is a popular antifungal antibiotic, but the exact way it works is still a matter of debate. Here, we used monolayers composed of phosphatidylcholine with ergosterol as a model of fungal lipid membranes to study drug incorporation from the aqueous phase and analyze the molecular reorganization of membranes underlying the biological activity of the antibiotic. The results show that the internalization of antibiotic molecules into membranes occurs only in the presence of ergosterol in the lipid phase. Comparison of images of solid-supported monolayers obtained by atomic force microscopy and lifetime imaging fluorescence microscopy shows the formation of intramembrane clusters of various sizes in the lipid phase, consisting mainly of antibiotic dimers and relatively large membrane pores (∼15 nm in diameter). The results reveal multiple modes of action of amphotericin B, acting simultaneously, each of which adversely affects the structural properties of the lipid membranes and their physiological functionality.

Amphotericin B (AmB) belongs to a class of polyene macrolide antibiotics and is used to treat deep-seated, life-threatening fungal infections^1,2^ (see Supplementary Figure S1 for a chemical structure). Paradoxically, despite several decades of medical use of AmB and its high effectiveness,^3^ the exact modes of its biological activity are still a matter of debate.^4,5^ Among the main mechanisms discussed is the destabilization of a structure of biomembranes, induced by the presence of the drug molecules in the lipid phase^6,7^ or as a consequence of the sequestration of sterols associated with the formation of extra-membraneous sterol-AmB bulk structures referred to as “sponges”.^8,9^ One of the consequences of the destabilization of the membrane structure would be the uncontrolled leakage of physiologically important ions through the biomembranes, disturbing the electrostasis of living cells. Another important mechanism underlying the antibiotic action of AmB is the formation of transmembrane pores that act as passive ion channels uncontrolled by any regulatory activity,^10^ thus also having a decidedly negative impact on physiological ion transport. Currently, there are two models of barrel-stave-like transmembrane ion channels formed by AmB molecules in the lipid phase: one composed of a single-length complex^11^ and the other consisting of a double-length channel formed by two stacked single-length structures, each present in a single leaflet of the lipid bilayer.^12^ In the opinion of many researchers, the results of the experiments supporting all of the individual modes of action of AmB concerning lipid membranes are convincing, thus making it difficult to sort out which molecular mechanism applies to the pharmacological effect of this antibiotic. In the present work, we designed experiments in combination with various imaging techniques to further address this interesting issue, important from the point of view of designing future formulations of this drug that are characterized by minimized toxic side effects for patients. We used a model system of monomolecular layers formed at the air–water interface with dioleoylphosphatidylcholine (DOPC) and ergosterol (Ergo), the main sterol of fungal cells, to study the incorporation of drug molecules into the membrane from the water phase.^13^ Monolayers deposited on a solid support were then subjected to functional imaging. The main advantage of using atomic force microscopy (AFM) was the very high spatial resolution of this technique, while the use of fluorescence lifetime imaging microscopy (FLIM) was advantageous due to the sensitivity of fluorescence lifetime parameters to the molecular organization of AmB.^14^ The use of various imaging techniques in studying the interactions of pharmacologically active molecules with membranes has proven to be highly effective in understanding the mechanisms of their biological activity.^15−17^ Surprisingly, the results obtained in this study demonstrate multiple modes of action of AmB on lipid membranes, all of which may account for the pharmacological effectiveness of this popular antibiotic.

Figure 1 presents a FLIM image of a film deposited from the single DOPC-Ergo monolayer formed at the air–water interface and exposed to AmB that was introduced into the water subphase. The fluorescence signal assigned to AmB shows the incorporation of the drug molecules into the lipid phase.^18^ The fluorescence signal of this polyene antibiotic can be effectively detected despite the low concentration in the monolayer system, thanks to the relatively high fluorescence quantum yield of AmB (∼4% in the dimeric form^19^) and the very high sensitivity of the detection system. The fluorescence emission spectra of AmB incorporated into monolayers are presented in the Supplementary Figure S2. The position of the fluorescence emission band on the wavelength scale suggests that AmB present in the system examined appears mostly in the form of dimers and small molecular aggregates.^14^ AmB fluorescence has not been detected in the same experiment carried out under identical conditions, except that Ergo molecules were not present in the lipid monolayer (see Supplementary Figure S3). This result confirms that the presence of sterols, in particular ergosterol, in the system is a prerequisite for the effective penetration of AmB from the aqueous phase into lipid membranes.^18^ Detailed analysis of the FLIM image (Figure 1) shows that AmB incorporated in the DOPC-Ergo membrane is not distributed homogeneously but rather self-associates into clusters characterized by dimensions in the range of 250–750 nm. The spatial resolution of the optical microscopic system used enabled the discrimination of structures with a minimum dimension of 226 ± 27 nm (see the Supporting Information for experimental details). It is therefore highly probable that exact dimensions of the smallest molecular structures formed are significantly lower. The time-resolved fluorescence analysis shows that AmB in the system appears in three molecular organization forms characterized by the fluorescence lifetimes τ_1_ = 0.35 ns (12%), τ_2_ = 1.8 ns (34%), and τ_3_ = 6.8 ns (54%), assigned to small aggregates (e.g., tetramers), parallel dimers, and antiparallel dimers, respectively.^14^ Importantly, the amplitudes of the AmB fluorescence lifetime components in the monolayer show that the antibiotic does not exist as a monomer and that dimers are the main forms of molecular organization in this system. This observation suggests that other molecules, including a sterol and possibly also a lipid, are involved in the formation of the relatively large AmB clusters observed, presumably serving as “molecular spacers” protecting against strong excitonic interactions between chromophores. Interestingly, such large structures are not distinguished in the AFM images of multicomponent monolayers (Figure 2). This suggests that some AmB structures separated by a relatively short distance can be resolved using the AFM technique but are recognized as single larger objects by the optical imaging. On the other hand, AFM-based images show the presence of relatively small structures (20–50 nm in diameter) distinctly differing in height above the level of the monolayer (∼3 nm, corresponding to the length of a single AmB molecule^20^). The pillar-like structures visible in the film topography are also distinctly different from the membranes in nanomechanical properties, as can be concluded from the sample imaging based on the AFM phase signal (see Supplementary Figure S4). In principle, such structures can be formed at the water–membrane interface and, thanks to their stability, remain preserved in the process of the deposition of monolayers on a solid support. The fact that such structures are not observed for lipid monolayers exposed to AmB but lacking Ergo (Supplementary Figure S3) suggests that sterol molecules are not only necessary for the internalization of AmB into the membrane but are also involved in the formation of the bulk structures detected using a topography imaging of the samples. More detailed analysis of the AFM images also reveals the appearance of relatively small depressions in the membrane, manifested as black dots (Figure 3). Such structures are typical for the lipid monolayers containing Ergo and exposed to AmB. The diameters of such structures (∼15 nm) are too large to be considered as single ion channels, but certainly, they have the potential to act as pores significantly affecting the membrane structure and disturbing physiological ion transport. Despite the fact that the diameters of pillar-like structures (Figure 2) are larger than those of pore-like structures (Figure 3), it is possible that both types of structures may have the same origin: namely, they are formed in the separation process from membrane clusters rich in AmB and Ergo. The observed differences in diameter can be related both to the molecular dynamics of the multicomponent membrane and the process of relaxation after the emergence of new structures as well as to the different detection of convex structures and cavities using the AFM technique.

**Figure 1:**
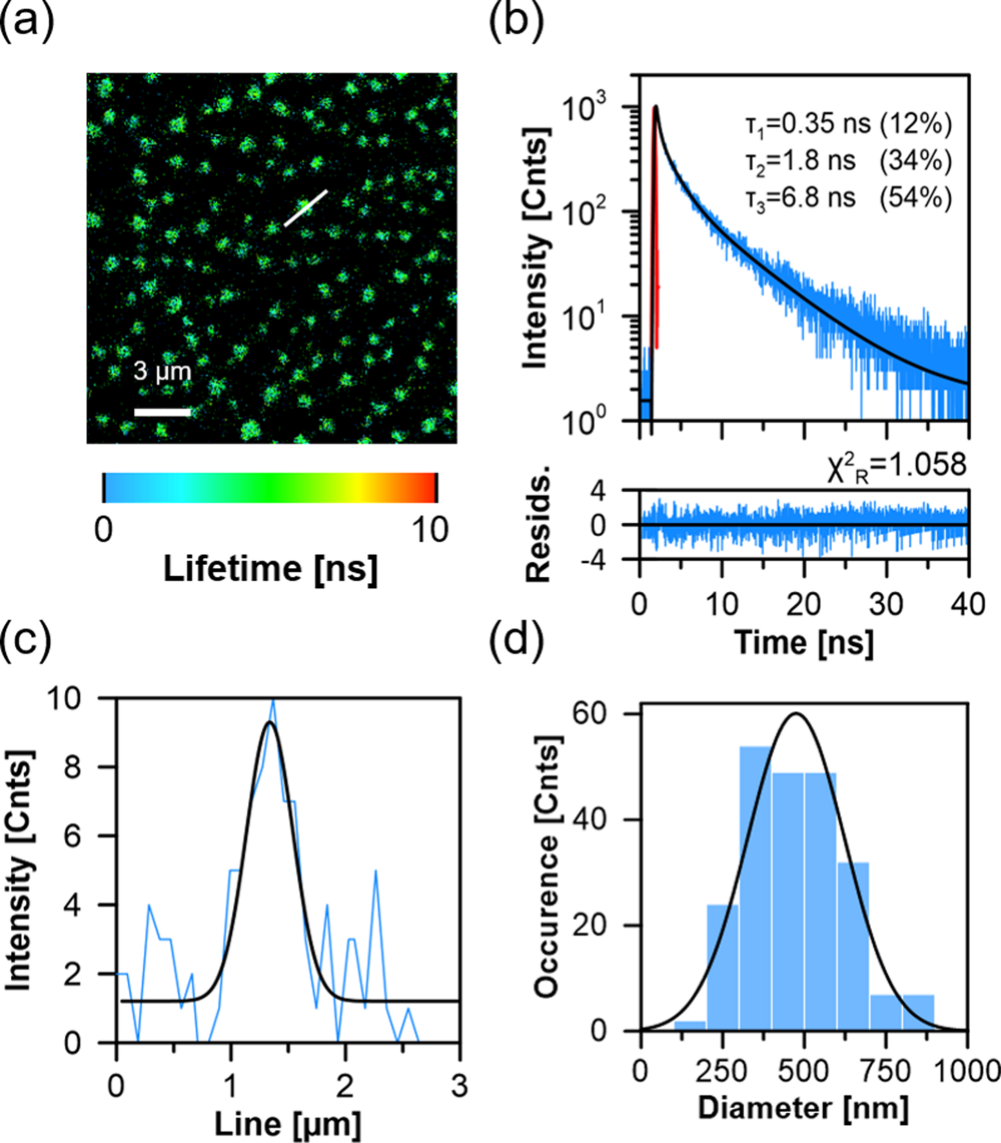
FLIM image and analysis of the film deposited from the monomolecular layer composed of DOPC and Ergo (7:3) exposed to AmB. (a) FLIM image, (b) fluorescence lifetime analysis based on all the photons detected during the imaging (IRF plotted in red), (c) cross-section analysis along the structure marked in panel (a) with white line, and (d) analysis of diameters of the fluorescence-emitting structures visible in panel (a) and another four images and based on the *fwhh* of fits to the signal as shown in panel (c). See the text for more information. The figure shows analyses of the exact same sample that was analyzed using AFM (results shown in Figure 2).

**Figure 2:**
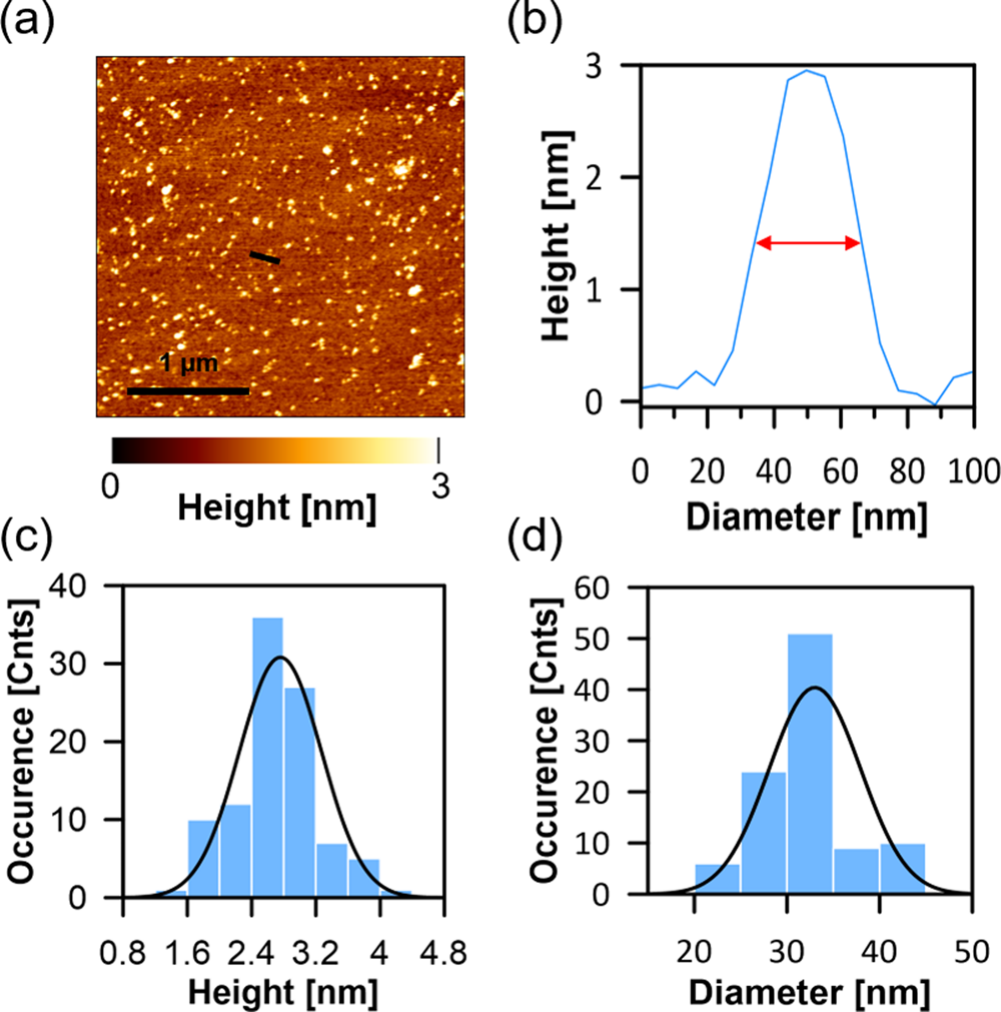
AFM image and analysis of the film deposited from the monomolecular layer composed of DOPC and Ergo (7:3) exposed to AmB. (a) AFM image, (b) cross-section analysis along the black line marked in panel (a), and analysis of height (c) and diameter (d) of the structures appearing as white dots in the image presented in panel (a), according to the methodology presented in panel b. The figure shows analyses of the exact same sample that was analyzed using FLIM (results shown in Figure 1).

**Figure 3:**
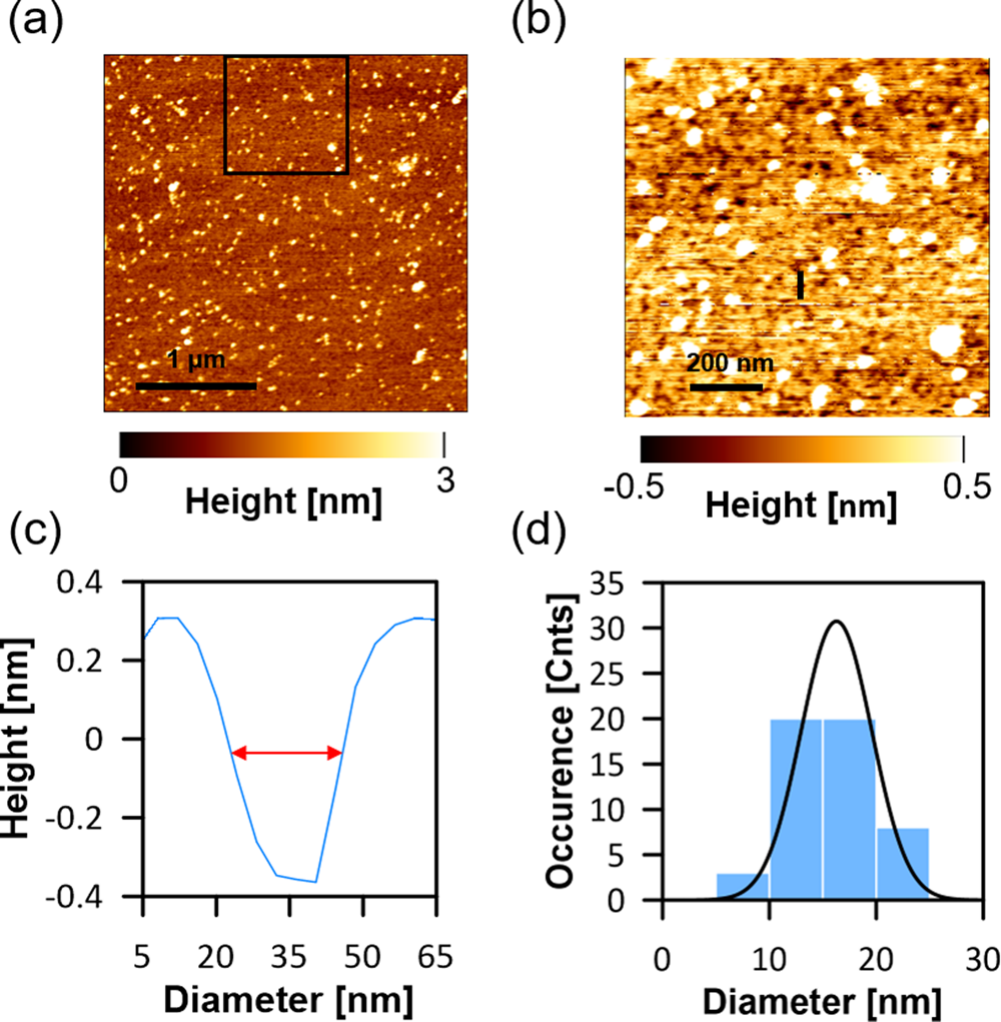
AFM image and analysis of the film deposited from the monomolecular layer composed of DOPC and Ergo (7:3) exposed to AmB. (a) AFM image, (b) detailed analysis of the image marked in panel a with black square, (c) cross-section analysis along the black line marked in panel (b), and (d) analysis of a diameter of the structures appearing as black dots in the image presented in panel (b), according to the methodology presented in panel (c).

The results of the analysis indicate the occurrence of various forms of molecular organization associated with the exposure of the lipid membrane containing Ergo to the action of AmB. First, AmB-related effects are observed only in sterol-containing lipid membranes. Such observations are in agreement with the results of the previous studies showing that sterols are necessary components of membranes that allow AmB molecules to penetrate into the lipid phase and ensure vertical orientation of the antibiotic molecules, along the axis perpendicular to the membrane plane.^18,21^ In the present study, two essentially different forms of AmB molecular organization in the membrane were observed, important from the standpoint of the structure–function relationship: intramembrane clusters separated from the lipid phase (20 to 50 nm in diameter) and ∼15 nm pores (see Figure 4 for a visualization). Importantly, AmB does not appear as a monomer but adopts more complex forms with the dimeric suborganization. It is concluded that the membrane components, presumably lipids and sterols, participate in the formation and stabilization of the observed structures. In our opinion, all these forms of molecular organization of AmB significantly influence the structural properties of biomembranes, which are important from a physiological point of view. Membrane phase separation, appearing as AmB-rich clusters, generates interfacial boundaries that may facilitate uncontrolled transmembrane transport of small molecules, including ions. An even greater risk of this process can be expected in the presence of membrane pores. The formation of extramembrane structures also seems to be important from a biological point of view. They likely represent molecular organization forms identified previously as sterol-rich AmB “sponges”. The results of model system studies presented in this work show that the presence of AmB in a lipid membrane containing sterols results in many consequences at the level of molecular organization and structural properties of membranes. Importantly, all of them may impair their biological functionality. This leads to the conclusion that the problem of the molecular basis of the pharmacological action of AmB is complex and is not limited to only one mechanism. Although the present study has revealed several effects of AmB on membranes, we are aware that due to the specificity of the techniques used and their resolution, several other potential mechanisms may not have been observed, e.g., the formation of very small ion channels. Apparently, the relatively complex and specific molecular structure of AmB itself modulates a whole series of interactions in the membrane environment, causing its significant reorganization, which manifests itself in many different ways depending on the research technique used.

**Figure 4:**
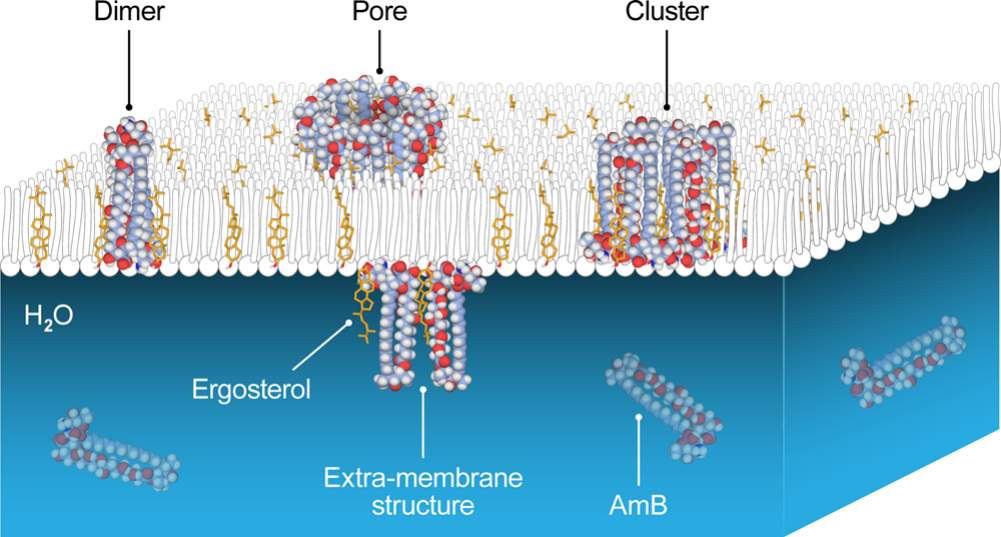
Model illustrating the process of internalization of AmB into the DOPC-Ergo monolayer from the aqueous subphase and the formation of various molecular structures.

## Supporting Information Available

Experimental details, chemical structure of amphotericin B, fluorescence emission spectrum of AmB incorporated into the lipid monolayer, AFM image based on the phase signal, AFM and FLIM images of the DOPC monolayers lacking Ergo and exposed to AmB, and isotherm of compression of the DOPC-Ergo monomolecular layer (PDF)
