# Supplementary material for: How Does the Antibiotic Amphotericin B Enter Membranes and What Does It Do There?
Source: J Phys Chem Lett. 2024 Apr 26;15(18):4823–7. doi: 10.1021/acs.jpclett.4c00496 (PMC11089563; doi:10.1021/acs.jpclett.4c00496)
Supplement: Supplementary file 1 — jz4c00496_si_001.pdf [file jz4c00496_si_001.pdf]

Supporting Information

How does the Antibiotic Amphotericin B Enter Membranes and What does it Do There?

Sebastian Janik[a], Rafal Luchowski[a,b], Ewa Grela[a,c], Wojciech Grudzinski[a], and Wieslaw I. Gruszecki[a,]*

[a] *Department of Biophysics, Institute of Physics, Maria Curie-Sklodowska University, 20-031 Lublin, Poland*

[b] *Department of Biophysics, Medical University of Lublin, 20-059 Lublin, Poland*

[c] *Division of Biophysics, Institute of Experimental Physics, Faculty of Physics, University of Warsaw, 02-093 Warsaw, Poland*

[*] corresponding author: wieslaw.gruszecki@umcs.pl

Experimental Section

Materials

Crystalline amphotericin B (AmB) was purchased from Cayman Chemical (USA). Directly before use, AmB was repurified chromatographically, as described in detail previously.[1] 1,2-dioleoyl-sn-glycero-3-phosphocholine (DOPC) was obtained from Avanti Polar Lipids (USA). Ergosterol was obtained from Merck (Germany). Methanol, 2-propanol and chloroform were purchased from POCH (Poland). Water used in experiments was purified by a Milli-Q Millipore system (Merck, Germany).

Preparation of monomolecular layers

Monomolecular layers were formed using a Teflon trough equipped with movable barriers, an injection port, and a magnetic stirrer. An integrated Langmuir and Langmuir-Blodgett system was purchased from KSV NIMA Instruments (Finland). A Wilhelmy tensiometer with an ashless filter paper (Whatman) as a surface pressure sensor was used to monitor surface pressure. Before starting the main part of the experiment, the DOPC:Ergo (7:3, mol:mol) monolayers were formed at the air-water interface and isotherms of compression were recorded (See Supporting Information Figure S5). A monolayer constituents were deposited at the air-water interface in 50 μl of a DOPC and Ergo solution prepared in a chloroform:methanol (9:1, v/v) solvent mixture. The monolayer compression began after 15 min. necessary for solvent evaporation. The barrier speed was constant at 10 mm/min. To examine the penetration of AmB from the water phase into the lipid monolayers, the films were compressed to 25 mN/m, and this surface pressure was stabilized automatically by the computer-controlled system. AmB was injected into the aqueous subphase, beneath the monomolecular layers, as a solution prepared in water/2-propanol mixture (6:4, v/v). A volume of 100 μl of AmB solution was injected into

~300 ml of water subphase. The concentration of AmB solution was adjusted to maintain the 1:1 ratio of molecules of AmB in the subphase and the total number of DOPC and Ergo molecules forming the lipid monolayers. The process of incorporation of AmB into the monolayers was manifested by a computer-controlled decompression of the films to maintain the surface pressure at 25 mN/m. After stabilization of this process (40 min.), the monomolecular layers were transferred to freshly cleaved Mica substrate by means of the Langmuir-Blodgett technique. The same constant surface pressure was automatically maintained by the system, also during the process of deposition of monomolecular films to a solid support. All experiments were performed at 25 °C. Single-component and multicomponent monomolecular layers formed with AmB, lipids, and sterols were characterized in detail in our previous studies.[2]

Atomic Force Microscopy

The monolayers were deposited on a freshly cleaved Mica surface at room temperature and then transferred to an AFM microscope. AFM scanning was carried out using JPK Nanowizard 3 system (Bruker, USA) in a non-contact mode (AC mode). RFESPA-190 cantilevers (Bruker, USA) with a nominal elastic constant of 35 N/m and a typical tip radius of 8 nm were used. The nominal resonance frequency of the cantilevers (provided by the manufacturer) was 190 kHz and the typical operating resonance frequency was 157.4 kHz. AFM images were scanned at 512 × 512 or 1024 × 1024 pixel resolution at 0.8 Hz. To avoid defects and imperfections, a regime of weak tip-sample interaction was applied during scanning by monitoring the tip dithering phase shift. AFM images were processed by subtracting the polynomial fit from each scan line independently then the height images were fed to cross sections using JPKSMP data processing software (Bruker, USA).

FLIM measurements

Time-resolved imaging experiments were conducted employing a MicroTime 200 microscope system purchased from PicoQuant (GmbH, Berlin, Germany). The samples were excited with 405 nm solid-state laser (Picoquant) with pulses characterized by a full width at half maximum (FWHM) of less than 90 ps. This wavelength of laser light was chosen to specifically excite the 0-0 vibrational maximum of the main electronic absorption band of AmB. The laser light beam was directed at the sample through an Olympus 60x objective with a numerical aperture of 1.2. The resulting fluorescence emission was collected by the same objective and transmitted to an avalanche photodiode detector (Excelitas Technologies) configured in a confocal mode. The detection efficiency of the detector was up to 70% at 500 nm, with a timing resolution down to <250 ps (FWHM). A pinhole diameter of 50 μm was utilized, and scattered light underwent filtration through a long-wavelength pass filter HQ430lp followed by a dichromatic mirror ZT405RDC, both sourced from AHF Analysentechnik.

The analysis of fluorescence components was executed utilizing SymPhoTime v. 2.8 software (PicoQuantGmbH, Berlin, Germany). This analytical process encompassed the identification of components based on characteristic fluorescence lifetime values (τ_i) derived from the emission intensity formula *I(t)*:

$$I(t) = \int_{-\infty}^{t} IRF(t') \sum_{i=1}^{n} A_i \, e^{-\frac{t-t'}{\tau_i}} dt' + Bkgr$$

Wherein, *t* represents time, *IRF(t')* denotes the instrument response function at a reference time *t'*, *n* is the number of exponentials, A_i signifies exponential prefactors, and *Bkgr* accounts for background correction.

Subsequently, intensities associated with each exponential component, reflecting non-zero contributions from distinct organizations of fluorophores, were computed:

$$I(i) = \tau_i \mathrm{A}_i$$

These intensities were indicative of the quantity of distinct molecular organizations of the antibiotic. Additionally, the amplitudes associated with the intensities were expressed as a percentage referenced to the total intensity.

Selected monomolecular film regions 10 x 10 μm or 20 x 20 μm were scanned at a resolution of 300 x 300 pixels. The optical resolution of the microscope, considering the specified objective parameters and excitation wavelength, enabled the discrimination of structures with a minimum dimension of 226 ± 27 nm.

The fluorescence signal from the bright spots observed in the microscopic images was extracted through the exit port of the microscope system and subsequently directed into a spectrograph for further analysis. To ensure the fidelity of the collected data and eliminate interference from Raman and Rayleigh scattered light, a long-wavelength pass filter (HQ430lp) was employed prior to detection (the same as used to record images). Spectral data were recorded using a spectrograph model SR 163 (Shamrock), which was equipped with a Newton 970 EMCCD camera from Andor Technology (detection efficiency of up to 95%). The camera was thermoelectrically cooled down to -60°C to minimize noise during data acquisition, thereby ensuring high-quality signal capture. The SR 163 spectrograph, employing a Czerny Turner design with a grating having 600 lines per millimeter, blazed at 500 nm, enabled the acquisition of fluorescence spectra across a spectral window of 250 nm. Data were recorded and processed using Solaris software, with exposure time (20 s) optimized to maximize signal-to-noise ratio. Subsequent statistical analyses were performed using established statistical tools implemented to Grapher program (Golden Software) to derive meaningful insights from the collected data.

All types of experiments, including AFM and FLIM imaging for different monolayers, were repeated at least 10 times and found to be reproducible.

REFERENCES

(1) Grela, E.; Wieczor, M.; Luchowski, R.; Zielinska, J.; Barzycka, A.; Grudzinski, W.; Nowak, K.; Tarkowski, P.; Czub, J.; Gruszecki, W. I. A mechanism of binding of an antifungal antibiotic amphotericin B to lipid membranes: An insight from combined single membrane imaging, micro-spectroscopy, and molecular dynamics. *Mol. Pharm.* **2018**, *15*, 4202−4213.

(2) Gagos, M.; Gabrielska, J.; Dalla Serra, M.; Gruszecki, W. I. Binding of antibiotic amphotericin B to lipid membranes: monomolecular layer technique and linear dichroism-FTIR studies. *Mol. Membr. Biol.* **2005**, *22* (5), 433-442.

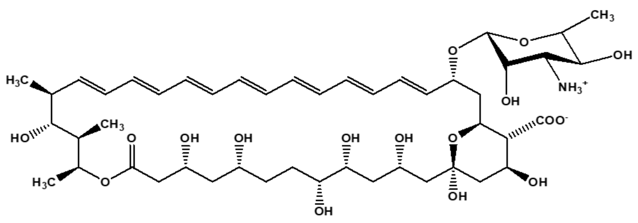

Figure S1 Chemical structure of amphotericin B.

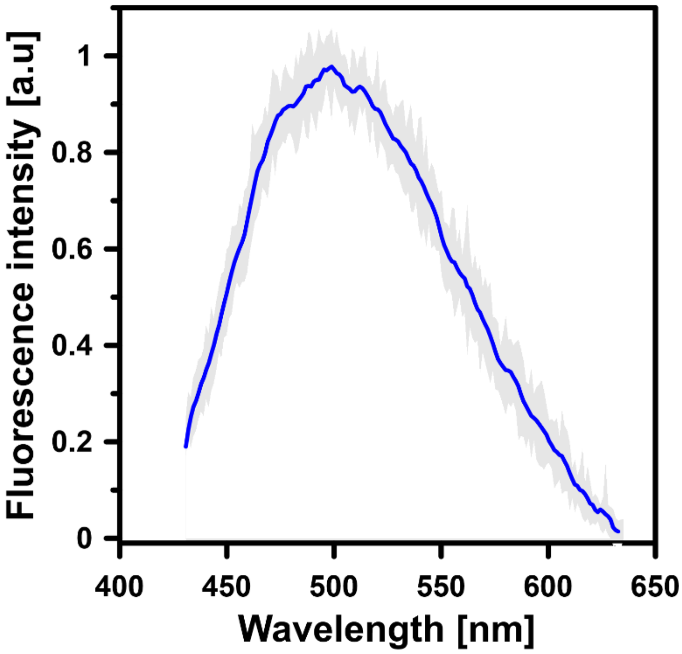

Figure S2 Fluorescence emission spectrum of AmB incorporated in a monomolecular layer composed of DOPC:Ergo (7:3, mol:mol). The spectrum (plotted in blue) represents the arithmetic mean of 16 individual, single-pixel spectra recorded from different fluorescence-emitting structures visible in the FLIM image. The gray area represents S.D. from the arithmetic mean. The spectra were recorded from a monolayer deposited on the mica surface.

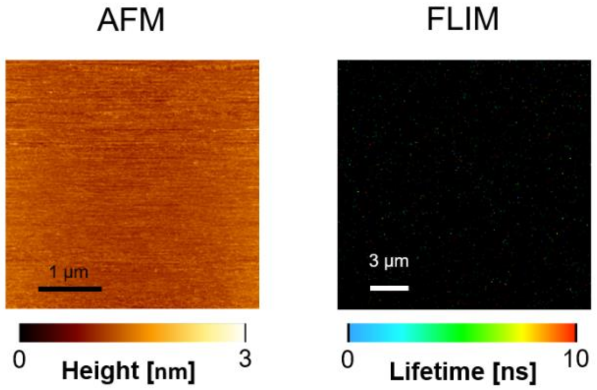

Figure S3 Images acquired with AFM and FLIM (indicated) of the same film deposited from a monomolecular layer formed with DOPC (without Ergo) and exposed to AmB. Note the lack of a signal assigned to AmB.

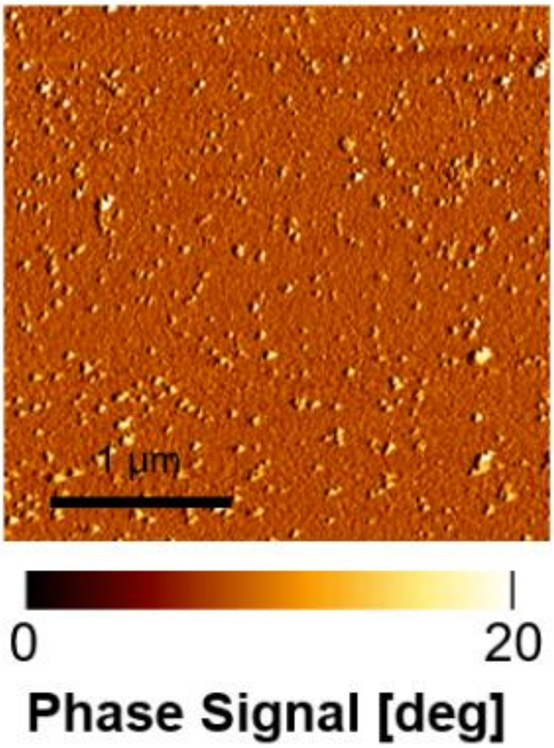

Figure S4 AFM image of the film deposited from the monomolecular layer composed of DOPC and Ergo (7:3, mol:mol) exposed to AmB. The image is based on a phase signal reflecting the mechanical properties of scanned objects.

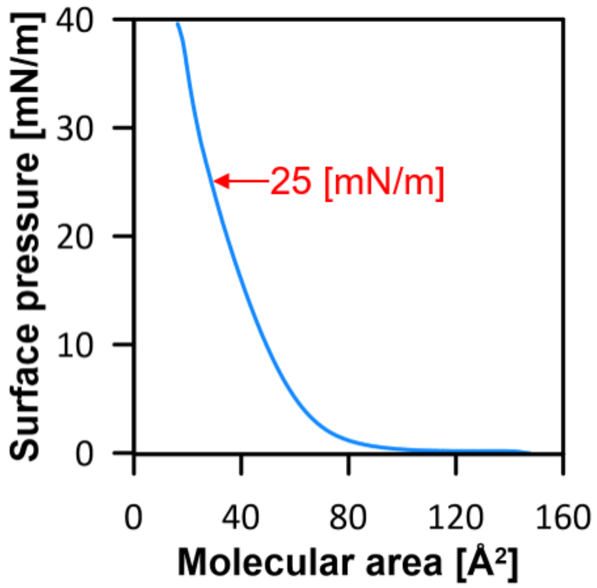

Figure S5 Surface pressure-mean molecular area isotherm of compression of a two-component monomolecular layer formed with DOPC:Ergo (7:3, mol:mol) at the air-water interface. In order to expose a monolayer to AmB, compression was stopped and stabilized at 25 mN/m, while AmB solution was injected into the aqueous subphase.
